# Gender similarities in the brain during mathematics development

**DOI:** 10.1038/s41539-019-0057-x

**Published:** 2019-11-08

**Authors:** Alyssa J. Kersey, Kelsey D. Csumitta, Jessica F. Cantlon

**Affiliations:** 1 Department of Brain and Cognitive Sciences, University of Rochester, Rochester, NY 14627 USA; 2 Department of Psychology, University of Chicago, Chicago, IL 60637 USA; 3 Department of Psychology, Carnegie Mellon University, Pittsburgh, PA 15213 USA

**Keywords:** Intelligence, Human behaviour

## Abstract

Some scientists and public figures have hypothesized that women and men differ in their pursuit of careers in science, technology, engineering, and mathematics (STEM) owing to biological differences in mathematics aptitude. However, little evidence supports such claims. Some studies of children and adults show gender differences in mathematics performance but in those studies it is impossible to disentangle intrinsic, biological differences from sociocultural influences. To investigate the early biology of mathematics and gender, we tested for gender differences in the neural processes of mathematics in young children. We measured 3–10-year-old children’s neural development with functional magnetic resonance imaging (fMRI) during naturalistic viewing of mathematics education videos. We implemented both frequentist and Bayesian analyses that quantify gender similarities and differences in neural processes. Across all analyses girls and boys showed significant gender similarities in neural functioning, indicating that boys and girls engage the same neural system during mathematics development.

## Introduction

Limited evidence for intrinsic, biological gender differences in mathematics ability has fueled debate about the underrepresentation of girls and women in STEM fields (science, technology, engineering, and mathematics). Some have suggested that girls and women are underrepresented in careers in STEM owing to biological differences.^1^ Biological sex differences manifest in aspects of brain function, particularly those related to neuroendocrinology,^2–4^ but many measures indicate that neural variability is a continuum wherein the brains of males and females reflect one heterogenous population rather than two distinct groups.^4^ In the domain of mathematics, the evidence for biologically based gender differences is weak because when gender differences are observed, the studies fail to differentiate intrinsic biological factors from sociocultural ones.^5–9^ Moreover, behavioral studies often find no gender differences in mathematical cognition in early childhood, and there are no prior functional neuroimaging studies of biological gender differences in mathematical cognition during early childhood.^5,8–13^ In order to understand the origins of mathematics ability, and whether there are any gender differences, it is important to ask whether boys and girls begin development with biological differences in mathematical processing. Here, we combine frequentist and Bayesian statistical approaches to test for gender similarities and differences in the neural processing of mathematics during early childhood. We use “gender” instead of “sex” throughout this manuscript, which accords with the relevant literature,^12–14^ and because we collected parental report of children’s gender and did not measure their chromosomes.

Although evidence for behavioral gender differences in mathematics is weak in older children, adolescents, and adults, it is important to consider when and how any differences might emerge. One possibility is that despite established gender similarities on behavioral tasks in early childhood,^5,10,12,13^ the underlying biological or neural processes could differ between boys and girls. For example, boys’ and girls’ incorrect responses could result from different neural processes (e.g., inefficient recruitment of math processing regions vs inefficient response selection mechanisms). Boys’ and girls’ error rates could yield the same levels on behavioral tests of mathematics but the biology that underlies the errors in each gender group could differ. Alternatively, boys and girls may show significant, widespread biological similarities in the neural processes of mathematics during early childhood. This outcome would be consistent with yet untested claims that boys and girls share a core biology for mathematical cognition.

To compare the neural processes underlying mathematics development, we used functional magnetic resonance imaging (fMRI) to measure neural activity in 3–10-year-old children while they watched video clips that targeted early childhood mathematics skills (e.g., counting, addition; see Methods for more details). In total, 104 children (55 girls) participated in one of three natural viewing tasks (2 published studies^15,16^ + 1 unpublished study, under review). Data were combined across natural viewing tasks by normalizing each subject to a within-task adult baseline using intersubject correlations.^17^ This approach yields 80% power to achieve a medium-effect size of *d* = 0.55, *p* < 0.05 for independent samples *t* tests.

Intersubject correlations were conducted by comparing each child with every other child and comparing each child with every adult within a comparison group (63 total adults, 25 women, who watched one set of video clips). This resulted in an index of ‘neural maturity’ (child-to-adult intersubject correlations) and an index of ‘neural similarity’ (child-to-child intersubject correlations). The majority of analyses focus on similarities and differences in ‘neural maturity’, which indicates how well-developed and adult-like each child’s brain is during mathematical processing.^15,16^ Neural maturity was calculated by conducting intersubject correlations^17^ of the neural timecourses across the entire video between children and adults in every voxel of the brain (see Methods for details). Thus, it assesses the degree to which children’s neural activity resembled that of adults who watched the same video and allows the data to be combined across studies in a meaningful way. Within-child comparisons of neural maturity calculated to women vs men revealed that children’s neural maturity did not statistically differ based on the gender of the adult comparison group (see Methods for details). Therefore, for each child, their measure of neural maturity is averaged across all adults who watched the same video.

Girls’ and boys’ neural maturity were statistically compared across five whole-brain analyses which test for differences in mean neural maturity, similarities in mean neural maturity, differences in variance of neural maturity, and differences in the rate of mathematics development. First, we conducted frequentist statistical tests of differences (two-sided, independent samples *t* tests) and similarities in neural maturity. Similarities in mean neural maturity were assessed using statistical equivalence statistics. Testing for statistical equivalence is critical for evaluating gender similarities because a null result from a *t* test only suggests that there is not enough evidence to conclude that a difference exists—to address this we conducted a statistical test of similarity, Schuirmann’s Test of Equivalence.^18^ This test uses two one-sided *t* tests to determine the likelihood that the mean difference between two groups falls within a specified similarity range (consistent with previous work that tested for similarities and differences in SAT scores, we used a similarity range of 2/3 of a standard deviation^19^). Complementary to this approach, we conducted a Bayes Factor analysis, which also allows for the interpretation of both significant differences and significant similarities. The Bayes Factor analysis weighs the evidence for an alternative hypothesis against the evidence for the null hypothesis by taking the ratio of the posterior probabilities for the two hypotheses (the Bayes Factor). Bayes Factors > 3 indicate substantial, interpretable evidence for the corresponding hypothesis, and Bayes Factors < 3 suggest that the evidence is only anecdotal. Following previous work,^13^ the prior for the alternative hypothesis of gender differences was the default Cauchy distribution centered on the prior for the null hypothesis with a width of 0.707. The prior for the null hypothesis was 0. To test for differences in neural variance between boys and girls across the whole brain, we used Levene’s Test of Variance. Some have claimed that differences in the upper and lower tails of the distributions drive gender differences.^20^ Variance thus is an important measure because previous work shows that gender differences in variance can exist even when mean performance is the same.^10,21^ Following the whole-brain analyses, we present more-detailed region-of-interest analyses on regions of the number processing network (bilateral intraparietal sulcus, bilateral inferior frontal gyrus, and anterior cingulate cortex), which were defined from an independent functional localizer scan. In a final analysis, we calculated intersubject correlations across children to obtain measures of within-gender and between-gender neural similarity. We then directly compared children’s neural similarity as calculated with children of the same and of the different gender. If gender differences in neural activity have a biological categorical origin rooted in childhood, these categorical differences should be evident in the brain. In contrast, if gender differences in neural activity do not originate from categorical differences in early childhood, there should instead be widespread similarities.

## Results

### Frequentist and Bayesian comparisons of neural maturity

**Fig. 1:**
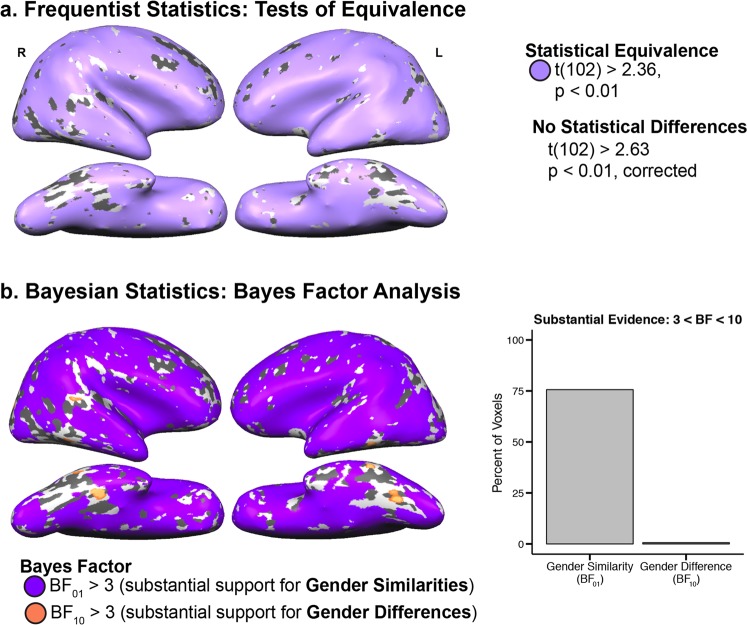
Whole-brain analyses. **a** Frequentist statistical tests of gender differences and similarities (light purple) of neural maturity. Using frequentist analyses (*t* tests), there are no regions showing significant gender differences at the standard threshold. **b** Whole-brain Bayes Factor analysis showing substantial evidence of gender differences (orange) and gender similarities (dark purple) of neural maturity. The plot to the right shows the percent of voxels across the brain that show substantial support for gender similarities and differences. The regions that show evidence of gender similarities are consistent across frequentist and Bayesian approaches

We used intersubject correlations to compare girls’ and boys’ temporal patterns of neural activity across the whole brain during the educational videos. First, we compared girls’ and boys’ overall neural maturity using frequentist statistics. A whole-brain *t* test revealed no differences in neural maturity between groups (threshold = *t*(102) ≥ 2.36, voxel-wise *p* ≤ 0.01, cluster corrected to *p* < 0.05, cluster threshold = 29 voxels). Next, gender similarities were assessed using Schuirmann’s Test of Equivalence.^18^ Findings of statistical equivalence would suggest that children’s neural processing of mathematics comprises one heterogenous group rather than two distinct gender groups. In fact, girls and boys showed statistically equivalent levels of neural maturity throughout the brain (Fig. 1a, light purple; minimum t(102) ≥ 2.36, maximum one-sided *p* ≤ 0.01), suggesting that the neural processing of mathematics develops at similar rates in boys and girls. In terms of differences in neural variance, Levene’s Test of Variance revealed one small region of right posterior parietal cortex where groups differed in variance but not in mean activation, with girls showing greater variance than boys (TAL peak: 18, −64, 43; F(1,102) ≥ 6.89, voxel-wise *p* ≤ 0.01, cluster corrected to *p* < 0.05, cluster threshold = 11 voxels, Supplementary Fig. 1). This variance cluster was small (15 voxels) and girls exhibited more variance than boys at equal neural amplitudes to boys, which did not result in a mean difference between groups in this region.

The pattern of large-scale statistical similarities between boys and girls from the frequentist analyses was replicated in the Bayesian analysis. In each voxel of the brain, the weight of evidence for the null and alternative hypotheses were indexed by the Bayes factor (B_01_ for the null hypothesis of gender similarities, B_10_ for the alternative hypothesis of gender differences). Bayes factors suggesting that the data provide substantial support of the hypotheses are displayed in Fig. 1b (B_01_ > 3, in purple indicating gender similarities, B_10_ > 3 in orange indicating greater neural maturity in girls; no cortical regions showed substantial support for greater neural maturity in boys). Less than 1% of voxels (0.8%) showed substantial or strong (B_10_ > 10) evidence of gender differences (Fig. 1b; 23.6% voxels did not substantially support either similarities or differences in the Bayesian analysis).

### Similarities in math processing networks

**Fig. 2:**
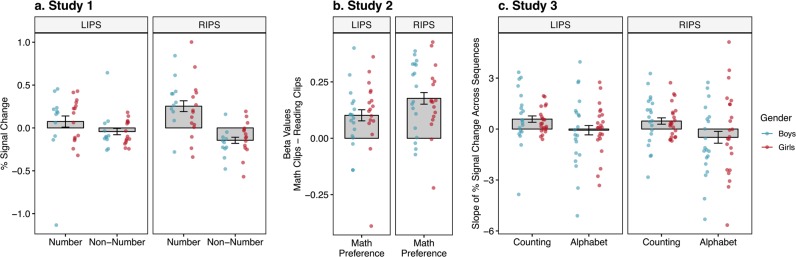
Number and math selectivity in the intraparietal sulci (IPS). Individual data for boys are shown in blue and for girls are shown in red. Error bars represent ±1 standard error of the mean. **a** Percent signal change for number clips vs non-number clips in Study 1—redrawn data^15^ (RIPS: t(25) = 4.01, *p* = 0.0005, 95% CI = 0.19–0.60, Cohen’s *d* = 0.79; LIPS: t(25) = 1.51, *p* = 0.26, 95% CI = −0.09–0.33, Cohen’s *d* = 0.23). **b** Preference for math clips vs non-math clips in Study 2—redrawn data^16^ (RIPS: t(34) = 6.86, *p* < 0.0001, 95% CI = 0.12–0.23, Cohen’s *d* = 1.16; LIPS: t(34) = 4.12, *p* = 0.0002, 95% CI = 0.05–0.15, Cohen’s *d* = 0.70). **c** Slope of % signal change across increasing counting vs alphabet sequences in Study 3 (higher slope = greater sensitivity to sequence; RIPS: t(42) = 3.21, *p* = 0.003, 95% CI = 0.35–1.56, Cohen’s *d* = 0.49; LIPS: t(42) = 2.55, *p* = 0.014, 95% CI = 0.14–1.17, Cohen’s *d* = 0.39). Independent *t* tests suggest no differences between girls and boys (max *t* value = 0.84, min *p* value = 0.41) and Bayes Factor analyses suggest anecdotal to substantial evidence for gender similarities (BF_10_ for gender differences: 0.30–0.47; BF_01_ for gender similarities: 2.14–3.25). Although, samples for each individual study are small, effects are consistent with the larger patterns reported in the following analyses

Importantly, across all three natural viewing tasks, children engaged numerical processes in the brain. Children showed number-selective neural activation in the intraparietal sulci (IPS) during the mathematics content in the educational videos (Fig. 2) consistent with previous fMRI research on numerical cognition in children and adults.^15,16^ Boys and girls showed equivalent mathematics-related neural responses (see Fig. 2). This is evidence that children engaged mathematical neural processes during the educational videos, and that boys and girls did so equally.

**Fig. 3:**
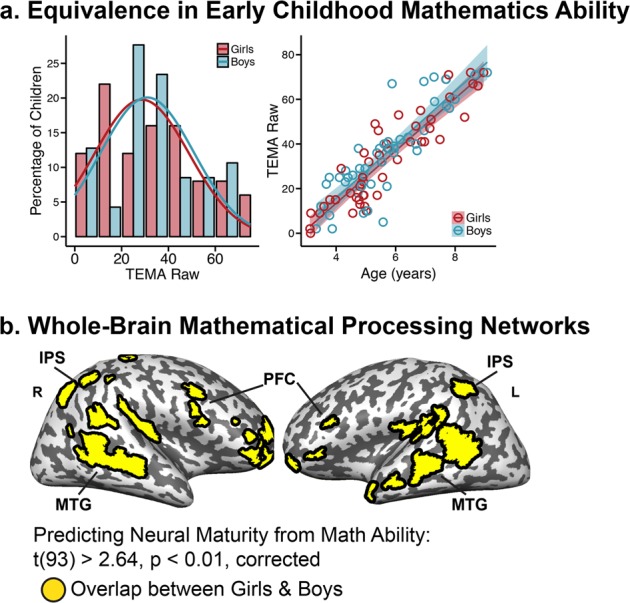
Gender similarities in math ability. **a** Left: distributions of TEMA-3 scores for girls (red) and boys (blue). Right: TEMA-3 scores increase with age for girls (red) and boys (blue), shaded regions around the line represent ±1 standard error of the mean. **b** Regions where boys and girls showed a relation between neural maturity and math ability. Importantly, no cortical regions showed an interaction between math ability and gender. Abbreviations: IPS = intraparietal sulcus, PFC = prefrontal cortex, MTG = middle temporal gyrus, R = right, L = left

Next, we compared the rate of mathematics development in boys and girls. Ninety-seven children completed the Test of Early Mathematics Ability to evaluate their mathematics skills (TEMA-3^22^; *n* = 50 girls, 3.12–8.96 years; 47 boys, 3.33–9.08 years). Math ability was statistically equivalent across children and did not show gender differences in mean ability or variance (Fig. 3a; *t* test: t(95) = 0.57, *p* = 0.57, girls’ mean = 33.62, boys’ mean = 35.96, 95% CI = −10.42–5.74; Tests of Equivalence: t_1_(95) = 3.84, *p* < 0.001, t_2_(95) = −2.70, *p* = 0.004; Test of Variance: F(1,95) = 0.29, *p* = 0.59, girls’ sd = 20.19, boys’ sd = 19.87; descriptively there were more girls than boys in the upper tail of the distribution: 14 girls and 10 boys). Nor did the relation between gender and math ability change across age (Fig. 3a; Regression of TEMA-3 on gender and age: *R*^2^ = 0.79, F(3,93) = 118.5, *p* < 0.0001, Gender: *b* = 1.22, t(93) = 0.163, *p* = 0.871; Age: *b* = 11.74, *t* = 13.97, *p* < 0.0001; Gender × Age Interaction: *b* = 0.26, *t* = 0.21, *p* = 0.84). These behavioral data were previously included as part of a larger behavioral study that showed no differences and statistical equivalence in math ability in this age group.^12^

We then identified mathematical processing networks by testing for regions that showed higher neural maturity in children with stronger math skills. Math ability, gender, and the interaction between math ability and gender were entered as predictors of neural maturity in a whole-brain regression. This regression revealed that math ability predicted neural maturity in both gender groups in the IPS, prefrontal cortex, and middle temporal gyrus (Fig. 3b; t(93) ≥ 2.63 voxel-wise *p* ≤ 0.01, cluster corrected to *p* < 0.05, girls: r(48) ≥ 0.36 with cluster threshold of 109 voxels, boys: r(45) ≥ 0.37 with cluster threshold of 69 voxels, see Supplementary Fig. 2 for separate maps). These regions are consistent with those that show a correlation between neural maturity and math ability when collapsed across gender (r(95) ≥ 0.26, *p* ≤ 0.01, corrected, Supplementary Fig. 2). No cortical regions showed significant interactions between math ability and gender (t(93) ≤ 2.63, threshold: voxel-wise *p* ≤ 0.01, cluster corrected to *p* < 0.05, threshold = 110 voxels), indicating that the relation between math ability and neural maturity does not depend on gender. In other words, mathematical processing networks develop at the same rate for girls and boys.

**Fig. 4:**
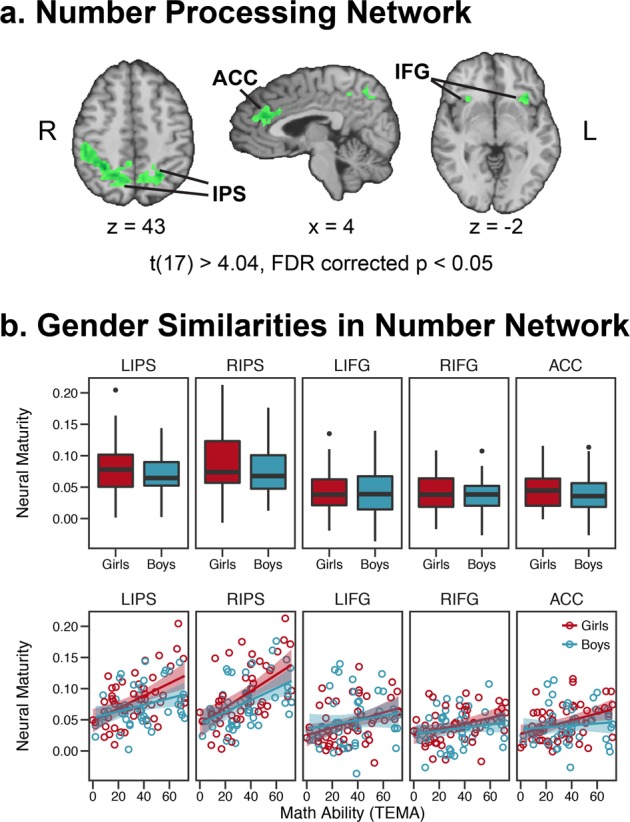
Region-of-interest analyses. **a** Number processing network identified from an independent localizer (number matching > face, shape, & word matching). **b** Mean neural maturity in the number network (top) and relation between neural maturity and math ability in the number network (bottom). Boxplot center line identifies the median, the upper whiskers extend from the 75th percentile to the 75th percentile + 1.5 interquartile range, the lower whiskers extend from the 25th percentile to the 25th–1.5 interquartile range. Outliers are those data points beyond the whisker ranges. Shaded regions around the lines in the scatterplots indicate ±1 standard error. Abbreviations: IPS = intraparietal sulcus, ACC = anterior cingulate cortex, IFG = inferior frontal gyrus, R = right, L = left

To visualize patterns of gender similarities, neural maturity was extracted from an independently-defined number processing network^15^ consisting of bilateral IPS, bilateral inferior frontal gyrus, and anterior cingulate cortex (number > face, shape, and word matching; Fig. 4a; t(17) ≥ 4.04, FDR corrected to *p* < 0.05). In accord with the whole-brain analyses, these regions showed statistical equivalence, not statistical differences, and no differences in variance (Fig. 4b; *t* tests: max t(102) = 1.08, *p* = 0.28; equivalence-tests: min t_1_(102) = 3.82, *p* = 0.0001; min absolute value of t_2_(102) = 2.33, *p* = 0.01; see Supplementary Table 1 for full statistics). Regression analyses revealed that math ability predicted neural maturity throughout the number processing network, particularly in the IPS, but did not interact with gender (interaction predictors: max t(93) = 1.42, *p* = 0.16, Supplementary Table 1). This shows that within key number processing regions of the brain, girls and boys show the same degree of development in mathematical processing.

### Whole-brain child-to-child similarity

**Fig. 5:**
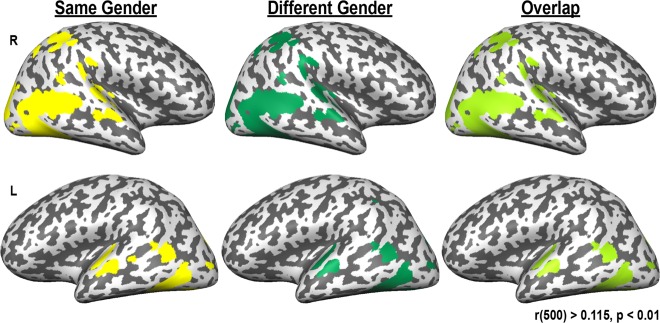
Child-to-child neural saimilarity. Average neural similarity (r(500) ≥ 0.115, *p* ≤ 0.01) when calculated across children of the same gender (yellow, column 1) and children of different genders (green, column 2). The third column shows the overlap of the first two columns in light green. Importantly, regions are identical across neural similarity calculated to children of the same gender and to children of different genders

Finally, we examined neural similarity in children of the same versus different genders. Intersubject correlations were calculated between children, resulting in maps of same-gender neural similarity (comparing girls with girls and boys with boys) and different-gender neural similarity (comparing girls with boys and boys with girls). To determine whether there were differences between same-gender versus different-gender similarity, each child’s whole-brain different-gender similarity map was subtracted from their same-gender similarity map. These difference maps were then subjected to a one-sample *t* test vs 0. This whole-brain *t* test revealed no regions that showed a difference in neural activity (threshold: t(103) ≥ 2.62, voxel-wise *p* ≤ 0.01, corrected to *p* < 0.05 with a cluster threshold of 19 voxels). Figure 5 shows average neural similarity calculated to children of the same gender (yellow), a different gender (dark green), and the overlap of those maps (light green; r(500) ≥ 0.115, *p* ≤ 0.01). The regions that showed strong neural similarity between children were identical for statistical comparisons of the same gender and different genders. This again indicates that children’s patterns of neural activity reflect one heterogenous group, rather than two distinct groups based on gender.

## Discussion

Across multiple neural analyses, we show that girls’ and boys’ brains function similarly during mathematical processing. We saw no evidence of gender differences in neural responses to mathematics content, neural responses during educational video viewing, or rates of neural development for mathematical processing in early childhood, and in fact we found statistical equivalence between boys and girls throughout the brain. Tests of statistical equivalence and a Bayes Factor analysis show gender similarities throughout the number processing network. Furthermore, boys’ and girls’ math abilities related to the rate of neural mathematics development in the same brain regions, and neural similarity was consistent across children of the same and of different genders.

Our results are consistent with the ‘Gender Similarities Hypothesis’, which argues that boys and girls function similarly in most areas of cognition.^10,23^ In particular, gender similarities in early childhood mathematics show, as proposed in the domain of spatial cognition,^14^ that gender differences in STEM fields in adults are not derived from intrinsic differences in children’s brains but likely from a complex environmental origin.

Any test of cognitive ability that shows gender differences faces the difficulty of disentangling biological factors from social ones. For instance, 4- to 7-year-old boys show an advantage over girls in tests of spatial skills, but parents also report more-spatial play with their boys compared with their girls,^14^ suggesting a possible sociocultural influence on gender differences in spatial cognition. Similarly, in math and science, teachers tend to show differential distributions of time spent encouraging students, praising students, and explaining concepts to students, with boys receiving more time than girls.^24–27^ This is important because teachers’ perceptions of children’s math ability predicts later math achievement scores.^28^ Parents’ expectations about their children’s success also correlate with children’s own self-concepts of their abilities and their performance on math tasks.^29,30^ A strong sociocultural influence on early childhood math achievement makes it difficult to tease apart intrinsic gender differences from sociocultural factors in older children and adults.^8,9^

Given the broad similarities between boys and girls, gender differences observed in STEM performance during adolescence or adulthood are unlikely to originate from early childhood differences in the brain or cognition. Although gender differences in STEM may emerge later in development or from interactions between STEM training and sexually dimorphic behaviors (e.g., differences in hormone levels following puberty),^6,31,32^ the findings of widespread gender similarities in boys’ and girls’ brains do not support claims of biological gender differences in childhood. Instead, the data show that the neural functions underlying mathematical cognition are similar between genders and represent one heterogeneous population rather than two categorical groups.

## Methods

### Participants

In total, 104 typically-developing 3- to 10-year-old children (55 girls) and 63 adults (35 women) participated in one of three studies. Age was statistically equivalent between girls and boys (t_1_(102) = 3.32, *p* = 0.0006, t_2_(102) = 3.43, *p* = 0.0004), and there were no differences in age variability, reflecting an even distribution of age across-gender groups (F(1,102) = 0.07, *p* = 0.79, girls’ sd = 1.65, boys’ sd = 1.63). Informed written consent was obtained from adult participants and parents of children, and informed written assent was obtained for children 7 years and older. All protocols were approved by the University of Rochester Research Subjects Review Board.

### fMRI paradigms

#### Study 1

Twenty-six 4- to 10-year-old children (15 girls; girls’ mean age = 6.93 years, boys’ mean age = 7.13 years; range = 4.32–10.80 years) and 20 adults (13 women; women’s mean age = 20.52 years, men’s mean age = 20.98 years; range = 18.9–25.4 years) successfully participated in Study 1. This paradigm consisted of a 20.3-min video containing clips from children’s educational television shows. Clips ranged from 12 to 176 s in length and were edited into a continuous movie. These data have been previously reported^15^ as the “natural viewing” task.

#### Study 2

Thirty-five 4- to 8-year-old children (17 girls; girls’ mean age = 6.61 years, boys’ mean age = 6.35 years; range = 4.08–8.67 years) and 23 adults (12 women; women’s mean age = 22.13 years, men’s mean age = 22.65 years; range = 18.44–28.09 years) successfully participated in Study 2. The 11.6-min video contained clips from children’s educational television shows. Clips ranged from 12.5 to 32.4 s in length and were edited into a continuous movie. These data have been previously reported^16^ as the “natural viewing” task.

#### Study 3

Forty-three 3- to 5-year-old children (23 girls; girls’ mean age = 4.54 years, boys’ mean age = 4.71 years; range = 3.12–5.96 years) and 20 adults (10 women; women’s mean age = 23.43 years, men’s mean age = 24.17 years; range = 20.15–31.55 years) successfully participated in Study 3. In this study, participants listened to pre-recorded audio tracks of someone counting or saying the alphabet. Short clips from child-friendly cartoons were presented on the screen during the sequences. Audio tracks were removed from the cartoons and were replaced with quieter, child-friendly instrumental music. Cartoon tracks were matched across sequences. Sequences were presented in 70 s blocks of 60 items presented at a rate of one item every 1.1–1.2 s. Fifteen blocks were presented throughout the experimental paradigm and were separated by 4-s of blackscreen. The scan began and ended with 12 s of blackscreen resulting in a total scan time of 19.2 min.

#### Number Localizer

Eighteen children from Study 1 (11 girls, 7 boys; girls’ mean age = 6.78 years, boys’ mean age = 7.47 years; range = 4.32–10.8 years) completed a traditional fMRI paradigm in which they compared pairs of stimuli (isolated images of faces, numbers, words or shapes) that were presented on the left and right sides of the screen. Participants reported whether they were the same or different by pressing a button only if the two stimuli matched. Half of the pairs presented were “matches”, whereas the other half were “non-matches”. Number comparisons were made across notation (i.e., dot array compared with Arabic numeral), face comparisons were made across a frontal shot and an oblique view, word comparisons were made across a word in all capital letters and the other in all lowercase letters, and shape comparisons were made across two shape images. Stimuli were presented in a blocked design. Each block consisted of three 2-s trials from the same condition, separated by a 2-s intertrial interval. Three blocks of each condition were semi-randomly presented throughout a run with 8 s of fixation between blocks. These data have been previously reported^15^ as the “traditional functional task”.

### fMRI session

Prior to the scanning sessions, children participated in a 30-minute training session in a mock scanner to familiarize them with the scanning environment and to practice remaining motionless. Children who completed the Number Localizer paradigm practiced the task prior to the MRI session. Adults received verbal instructions prior to scanning and did not participate in a training session. During the scan, children’s heads were secured with medical tape, headphones, and foam padding, and adults’ heads were secured with headphones and foam padding.

### MR parameters

Whole-brain BOLD imaging was conducted on a 3-Tesla Siemens MAGNETOM Trio scanner with a 12-channel head coil at the Rochester Center for Brain Imaging. High-resolution structural T1 contrast images were acquired using a magnetization prepared rapid gradient echo pulse sequence at the start of each session (repetition time (TR) = 2530 ms, echo time (TE) = 3.44 ms, flip angle = 7, field of view (FOV) = 256 mm, matrix = 256 × 256, 192, 176, or 160 slices depending on head size, 1 × 1 × 1 mm sagittal left-to-right slices). An echo-planar imaging pulse sequence with online motion correction was used for T2*contrast (TR = 2000 ms, TE = 30 ms, flip angle = 90 degrees, FOV = 256 mm, matrix 64 × 64, 30 axial oblique slices, parallel to the AC-PC plane, voxel size = 4 × 4 × 4 mm). The primary paradigms from Studies 1, 2, and 3 were 610 volumes, 348 volumes, and 567 volumes, respectively, and the Number Localizer paradigm was distributed over two to four functional runs of 132 volumes each.

### Preprocessing

fMRI data were analyzed in BrainVoyager^33^ using in-house scripts drawing on the BVQX toolbox. Data from previously published studies were analyzed as originally reported for consistency.^15,16^ For the Number Localizer and Study 1, which were collected during the same scanning session, the first six TRs of each run were discarded prior to analysis to allow for signal equilibration. For Studies 2 and 3, the first two TRs of each run were discarded. Functional data were registered to high-resolution anatomy images for each participant in native space. Preprocessing consisted of slice scan time corrected (cubic spline interpolation), motion correction with respect to the first volume in the run, and linear trend removal in the temporal domain (cutoff: two cycles within the run). A Gaussian spatial filter with an 8 mm full-width at half-maximum was applied to each volume for Study 1,^15^ and a 6 mm full-width at half-maximum was applied to each volume for Studies 2^16^ and 3. The functional data from the Number Localizer were not smoothed. Adult and child echo-planar and anatomical volumes were then normalized into Talairach space^34^ using piecewise affine transformation based on manual identification of anatomical landmarks. Analyses were performed on processed data in Talairach space. Average framewise displacement^35,36^ was regressed across the brain for each child to control for sudden changes in volume-to-volume head motion.

### fMRI data analyses

*Neural maturity:* neural data were analyzed using an intersubject correlation approach.^15–17^ Between-group intersubject correlations were performed by using the full timecourse of each voxel for each child as a predictor for activation of the corresponding voxel in each adult brain. Functional data from each child were then correlated with that of each adult from the same study to produce paired r-maps. Paired r-maps represent the neural similarity of each child compared with each adult in every voxel of the brain. A single, average brain map was then calculated to represent the neural similarity of each child to all adults. This map is referred to as a map of “neural maturity” because it shows how “adult-like” each child’s neural timecourse appears. To ensure that these similarity maps were not confounded by any adult gender differences prior to averaging, we compared neural maturity calculated to women vs men. For each child, we conducted an Independent Samples *t*-test to compare neural maturity when calculated to adults of the same vs different gender as the child. This resulted in a map of between-group *t* values for each child. To determine whether there were any differences that were consistent at the group level, the absolute value of these individual-level between-group *t* value maps were then subjected to a one-sample *t* test vs a critical *t* value of 2.08. The critical value represents the *t* value at which a difference could be considered significant given the smallest sample of adults (*n* = 20 for studies 1 and 3; t(18) = 2.08, *p* = 0.05). If there were a significant difference between neural maturity calculated to adults of the same vs different gender as the child, the one-sample *t* test should reveal a positive and significant effect, which would indicate that the absolute values of the individual-level between-group *t* values are significantly above 2.08. Instead, the whole-brain one-sample *t* test revealed that at the group level, the absolute values of the individual-level between-group *t* values were significantly below the critical *t* value of 2.08 (one-sample *t* test of absolute value of individual-level between-group *t* values vs critical *t* value of 2.08: t(102) ≤ −2.62, *p* ≤ 0.01). In other words, the majority of individual-level between-group t values were less than the critical *t* value of 2.08 (range of average absolute value of individual-level between-group *t* values across voxels: 0–1.90). This indicates that overall, the differences between neural maturity when calculated to adults of the same vs different gender were not different, so neural maturity was not biased by the gender of the adult.

#### Neural similarity

To compare how similar children were to each other, intersubject correlations were calculated across children following the same procedures as for calculating neural maturity. This resulted in two maps for each child: one representing the average neural similarity of a child to children of the same gender and one representing the average neural similarity of a child to children of the different gender. To compare neural similarity within and across gender, these maps were then subtracted from each other (within-gender similarity−across-gender similarity) and these difference maps were subjected to a one-sample *t* test vs 0.

#### Number Localizer

Functional data collected during the traditional fMRI paradigm were analyzed using a general linear model (random effects analysis). Experimental events (duration = 10 s) were convolved with a standard dual gamma hemodynamic response function. There were four regressors of interest (corresponding to the four stimulus categories), one regressor for button press, and six regressors of no interest (corresponding to the motion parameters obtained during preprocessing).

#### Region-of-interest (ROI) analyses

Data were extracted from number network ROIs using MATLAB. Analyses were then conducted using R (version 3.3.1) and R-Studio (version 0.99.902). Independent samples *t* tests were conducted using the “*t* test” function assuming equal variance. Tests of equivalence were conducted using the “TOSTtwo.raw” function from the “TOSTER” package (upper and lower bounds set to ±0.667 × standard deviation of the entire group; alpha = 0.05). This function returns two *t* values (*t*_1_ and *t*_2_). For statistical equivalence, both *t* values must be statistically significant. Statistical equivalence is rejected if either *t*_1_ or *t*_2_ does not reach significance. Levene’s Test of Variance was carried out using the “leveneTest” function in the “car” package. Regression analyses were conducted using the built-in “lm” function.

### Assessment of math ability

Math skills were evaluated by administering the TEMA-3^22^ to participants aged 8 and younger. The TEMA-3 tests a variety of math concepts and is standardized for 3–8-year-old children.

### Reporting summary

Further information on research design is available in the Nature Research Reporting Summary linked to this article.

## Supplementary information

KerseyCsumittaCantlon_SupplementalMaterial

Reporting Summary

## Data Availability

These data sets are available from the corresponding author on reasonable request.

## Code availability

All code for R and MATLAB are available from the corresponding author on reasonable request.
