# Supplementary material for: Gender similarities in the brain during mathematics development
Source: NPJ Sci Learn. 2019 Nov 8;4:19. doi: 10.1038/s41539-019-0057-x (PMC6841948; doi:10.1038/s41539-019-0057-x)
Supplement: Supplementary file 1 — KerseyCsumittaCantlon_SupplementalMaterial [file 41539_2019_57_MOESM1_ESM.pdf]

Supplementary Figures

Supplementary Figure 1 shows the results of Levene's Test of Variance as reported in the main text.

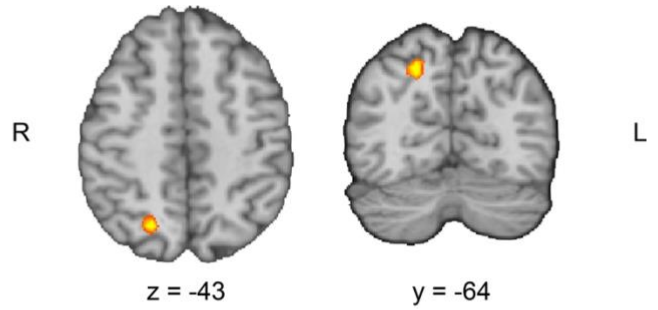

Supplementary Figure 1. No regions showed a difference in mean neural maturity. There were also no regions where boys show more variance than girls. Neural maturity was more variable in girls than boys in one small cluster of right posterior parietal cortex. (TAL peak: 18, -64, 43; $F(1,102) \geq 6.89$, $p \leq 0.01$, cluster corrected to $p < 0.05$ with a threshold of 11 voxels). This region did not differ in mean activation between boys and girls. No other region showed a difference in variance.

Supplementary Figure 2 shows the results of correlations between neural maturity and math ability for girls and boys.

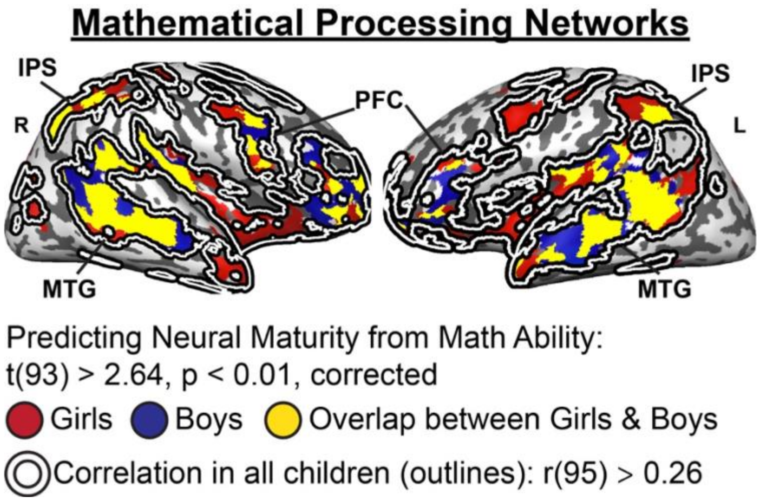

Supplementary Figure 2. Regions where math ability correlated with neural maturity in girls (red), boys (blue), and all children (outlines). The yellow regions show the overlap of the results for girls and boys and the same as those regions shown in Figure 2B in the main text. Non-overlapping regions did not show significant differences between girls and boys.

Supplementary Table

Supplementary Table 1 reports all statistics for the Region of Interest Analyses conducted on neural data from the number work as reported in the main text.

Supplementary Table 1. Statistics for Region of Interest (ROI) Analyses. Degrees of freedom are reported in parentheses for each test. sd = standard deviation. LIPS/RIPS = left/right intraparietal sulcus, LIFG/RIFG = left/right inferior frontal gyrus, ACC = anterior cingulate cortex. *indicates statistical significance ($p < 0.05$)

Statistical Analyses												
ROI	T-Test (Girls v Boys)				Equivalence Test				Variance			
	t(102)	p	Girls' mean	Boys' mean	t_1(102)	p_1	t_2(102)	p_2	F(1,102)	p	Girls' sd	Boys' sd
LIPS	1.08	0.28	0.080	0.072	4.46	0.00001*	-2.33	0.01*	1.85	0.18	0.041	0.032
RIPS	0.84	0.4	0.085	0.078	4.22	0.00003*	-2.56	0.0059*	1.09	0.30	0.050	0.040
LIFG	-0.44	0.66	0.044	0.047	3.82	0.0001*	-2.94	0.002*	1.28	0.26	0.033	0.040
RIFG	1.04	0.3	0.042	0.036	4.43	0.00001*	-2.34	0.01*	0.42	0.52	0.029	0.027
ACC	0.84	0.4	0.046	0.041	4.22	0.00003*	-2.55	0.006*	0.001	0.98	0.029	0.030

Regression Analyses												
ROI	F(3,93)	p	R^2	Gender			Math Ability (TEMA-3)			Interaction		
				Beta	t(93)	p	Beta	t(93)	p	Beta	t(93)	p
LIPS	8.54	0.00005*	0.22	0.005	0.35	0.73	0.001	4.32	0.00004*	-0.0005	-1.42	0.16
RIPS	12.54	< 0.00001*	0.29	0.0002	0.01	0.99	0.0013	4.94	< 0.00001*	-0.0003	-0.94	0.35
LIFG	2.47	0.066	0.07	0.01	0.69	0.49	0.0006	2.37	0.020*	-0.0003	-0.69	0.49
RIFG	2.81	0.044*	0.08	0.002	0.14	0.89	0.0004	2.44	0.016*	-0.0002	-0.72	0.47
ACC	2.93	0.038*	0.09	0.008	0.66	0.51	0.0005	2.73	0.008*	-0.0004	-1.35	0.18
